# miRNA-Dependent Control of Homeostatic Plasticity in Neurons

**DOI:** 10.3389/fncel.2019.00536

**Published:** 2019-12-05

**Authors:** Sandra Dubes, Alexandre Favereaux, Olivier Thoumine, Mathieu Letellier

**Affiliations:** ^1^University of Bordeaux, Interdisciplinary Institute for Neuroscience, UMR 5297, Bordeaux, France; ^2^CNRS, Interdisciplinary Institute for Neuroscience, UMR 5297, Bordeaux, France

**Keywords:** homeostatic plasticity, miRNA–microRNA, synaptic scaling, protein translation, membrane excitability, synaptic strength, synaptic plasticity

## Abstract

Homeostatic plasticity is a form of plasticity in which neurons compensate for changes in neuronal activity through the control of key physiological parameters such as the number and the strength of their synaptic inputs and intrinsic excitability. Recent studies revealed that miRNAs, which are small non-coding RNAs repressing mRNA translation, participate in this process by controlling the translation of multiple effectors such as glutamate transporters, receptors, signaling molecules and voltage-gated ion channels. In this review, we present and discuss the role of miRNAs in both cell-wide and compartmentalized forms of homeostatic plasticity as well as their implication in pathological processes associated with homeostatic failure.

## Introduction

Neurons employ a variety of homeostatic mechanisms to maintain network activity within physiological ranges in response to a wide range of remodeling events. These include for instance the assembly of synaptic circuits during development, learning and memory, or the pathological loss of synapses associated with neurodegenerative disorders (Hengen et al., 2013; Keck et al., 2013; Vitureira and Goda, 2013; Fernandes and Carvalho, 2016; Turrigiano, 2017). Depending on the situation, such homeostatic mechanisms may involve different signaling pathways, act on various physiological parameters, and operate on multiple time and space scales (reviewed in Turrigiano, 2012; Vitureira et al., 2012; Fernandes and Carvalho, 2016). It is therefore not surprising that the failure of neuronal homeostasis can impact physiological processes such as memory consolidation and synaptic circuit refinement (Mrsic-Flogel et al., 2007; Hengen et al., 2016; Diering et al., 2017), can contribute to epilepsy (Swann and Rho, 2014) and to various neurological disorders (Ramocki and Zoghbi, 2008; Dickman and Davis, 2009; Wondolowski and Dickman, 2013; Nelson and Valakh, 2015; Penn et al., 2017).

One important feature shared among the multiple forms of homeostatic plasticity is that they are slow as compared to Hebbian forms of plasticity, i.e., long-term potentiation (LTP) or depression (LTD), in which synaptic strengths are rapidly and durably potentiated or depressed, respectively. Homeostatic plasticity usually develops over the course of several hours, and up to several days, and relies on the synthesis of new proteins which regulate key physiological parameters (Turrigiano, 2012; Fernandes and Carvalho, 2016). Proteins as diverse as glutamate receptors (e.g., AMPARs), scaffolding proteins (e.g., PSD-95, PICK1), voltage-gated ion channels (e.g., P/Q-type calcium channels), kinases (e.g., CAMKIIβ, PKA), cell-adhesion molecules (e.g., β3-integrins) or soluble factors (e.g., TNFα, retinoic acid, BDNF) contribute to homeostatic plasticity through the regulation of synaptic efficacy, synapse number, and/or membrane excitability (reviewed in Turrigiano, 2012; Fernandes and Carvalho, 2016). So far, several studies have uncovered the role of activity-dependent mRNA transcription of immediate early genes like Plk2, Homer1a, Arc, and Narp (Shepherd et al., 2006; Seeburg et al., 2008; Chang et al., 2010; Gao et al., 2010; Diering et al., 2017) and the contribution of transcription regulators such as MSK1, MeCP2, and CaMKIV (Ibata et al., 2008; Blackman et al., 2012; Correa et al., 2012; Qiu et al., 2012). In contrast to transcriptional studies, a potential contribution of mechanisms regulating *de novo* protein synthesis at the post-transcriptional level such as mRNA translation and/or stability is just emerging (Fernandez-Moya et al., 2014; Kosik, 2016).

**FIGURE 1:**
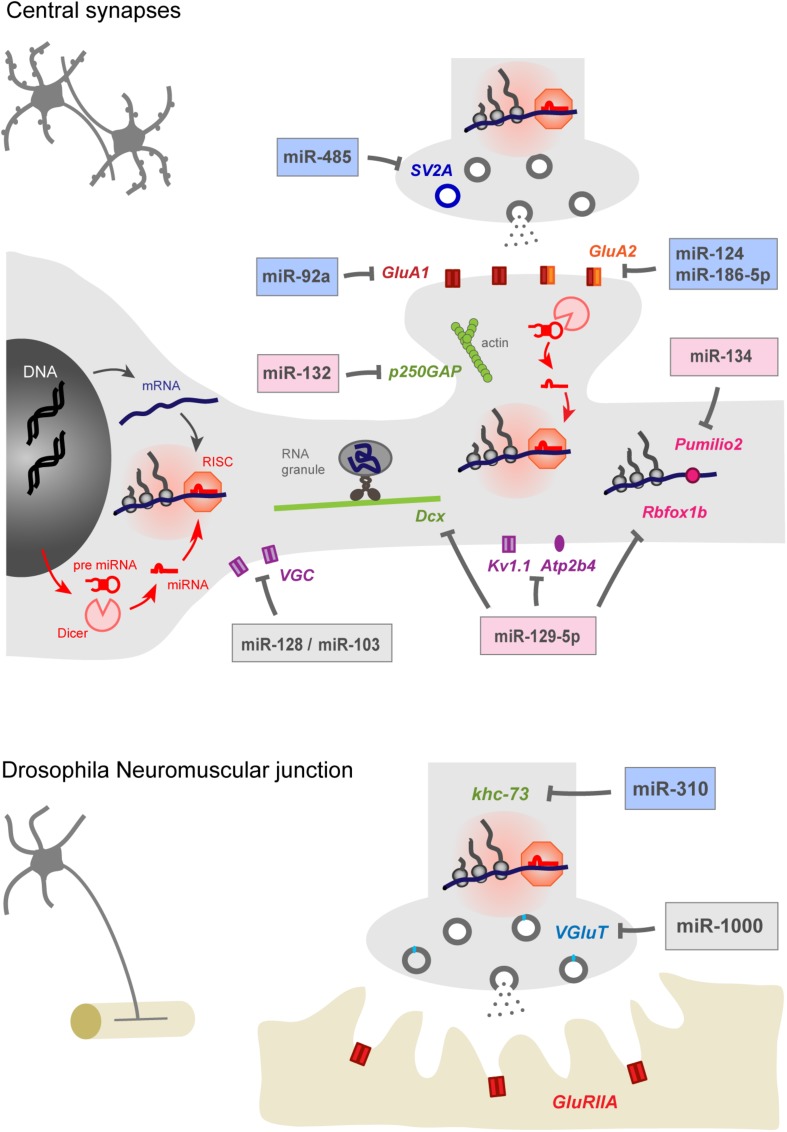
miRNAs contribute to homeostatic plasticity by controlling multiple effectors at central synapses of rodent models **(top)** and at the drosophila neuromuscular junction **(bottom)**. Identified miRNAs target presynaptic proteins regulating neurotransmitter release, post-synaptic AMPAR subunits, cytoskeleton-related proteins, voltage gated ion channels, calcium pumps, and RNA-binding proteins. miRNAs may repress protein translation at the cell body or in pre- or post-synaptic compartments, thereby providing autonomy to subcellular compartments and regulating appropriate physiological parameters, such as pre- and post-synaptic strengths and membrane excitability. miRNAs involved in the homeostatic up- or down-regulation of neuronal activity are highlighted in blue and pink while those showing bidirectional regulations are indicated in gray. Note that the schematic for rodent central synapses summarizes collective data from several neuronal types (see Table 1 for details).

Among the actors that may be involved in these processes, microRNAs (miRNAs) appear as important regulators of homeostatic plasticity in the nervous system. These small non-coding RNAs are highly enriched in the brain where they regulate a very large number of genes and shape transcriptomic diversity across regions (Filipowicz et al., 2008; Friedman et al., 2008; Soula et al., 2018). miRNAs are first synthesized in the nucleus then loaded in the RNA induced silencing complex (RISC), where they hybridize to the 3′ UTR of target mRNAs and inhibit protein synthesis through translational repression or destabilization of the transcript (Figure 1). The sequence involved in miRNA–mRNA interaction is called the “seed” region and is composed of the nucleotides 2–8 of the 5′ region of the miRNA (Bartel, 2009). Due to the small size of the “seed” region and the length of 3′ UTRs, the translation of a given mRNA is often under the control of multiple miRNAs while individual miRNAs can regulate the expression of dozens, if not 100s, of genes (Friedman et al., 2008). Loss of function approaches targeting individual miRNAs or their maturation through the endoribonuclease Dicer (Giraldez, 2005; Kim et al., 2007; Cuellar et al., 2008; Störchel et al., 2015; Fiorenza et al., 2016) have unveiled a contribution of the miRNA system in most aspects of neuronal development and plasticity, including neuronal differentiation and survival, neurite growth, synapse development, and plasticity (Kosik, 2006; Fineberg et al., 2009; Follert et al., 2014; Hu and Li, 2017; Tien and Kerschensteiner, 2018). In comparison with the regulation of transcription, which is spatially restricted to the nucleus, miRNAs provide an additional layer of regulations to finely tune in time and space protein synthesis in remote subcellular compartments such as synapses, and help cells adapt to their complex environment (Figure 1).

In this review, we present recent advances showing the contribution of several miRNAs in both cell-wide and compartmentalized forms of homeostatic plasticity through the regulation of the translation of multiple effectors (Figure 1 and Table 1). We first focus on homeostatic plasticity mechanisms that are regulated by miRNAs at the pre and post-synaptic levels, then discuss the impact of miRNAs on experience-dependent homeostatic synaptic plasticity (HSP) and neuronal excitability. Finally, we discuss several important questions that remain to be addressed, including the local versus global miRNA regulation and the implication of miRNAs in neuronal diseases.

**TABLE 1:** miRNAs involved in homeostatic plasticity and associated with neurological disorders.

	**miRNAs involved in homeostatic plasticity**	**Target(s)**	**Homeostatic plasticity paradigm(s)**	**Possible associated neurological disorder(s)**
Neurotransmitter release	miR-485 (Cohen et al., 2011) *Rat hippocampal cultures*	SV2A	Activity elevation (BIC + 4-AP/5 days)	Traumatic brain injury (Redell et al., 2009) Alzheimer (Lau et al., 2013)
	miR-1000 (miR-137 ?) (Verma et al., 2015) *Drosophila visual system*	VGlut	Dark rearing/constant light rearing	Rett syndrome (Szulwach et al., 2010) Alzheimer (Geekiyanage and Chan, 2011) Schizophrenia (Kwon et al., 2013) Rett syndrome (Cheng et al., 2014)
Post-synaptic receptors	miR-92a (Letellier et al., 2014) *Rat hippocampal cultures*	GluA1	Activity deprivation (TTX + AP5/4 h)	Rett syndrome (Urdinguio et al., 2010; Cheng et al., 2014) Autism spectrum disorder (Talebizadeh et al., 2008) Amyotrophic lateral sclerosis (Campos-Melo et al., 2018) Alzheimer (Patrick et al., 2017) Traumatic brain injury (Redell et al., 2009)
	miR-124 (Hou et al., 2015; Gilbert et al., 2016) *Rat hippocampal cultures*	GluA2	Activity deprivation (TTX + APV/15 h; TTX 24 h)	Huntington (Packer et al., 2008) Alzheimer (Lau et al., 2013; Gilbert et al., 2016) Epilepsy (Peng et al., 2013) Fragile-X syndrome (Xu et al., 2011) Rett syndrome (Urdinguio et al., 2010)
	miR-186-5p (Silva et al., 2019) *Rat hippocampal cultures*	GluA2	Activity deprivation (GYKI-52466 + MK-801/24 h)	Alzheimer (Kim et al., 2016) Autism spectrum disorder (Sarachana et al., 2010) Rett syndrome (Urdinguio et al., 2010; Cheng et al., 2014) 22q11.2 deletion syndrome (Stark et al., 2008)
	miR-218 (Rocchi et al., 2019) *Rat hippocampal cultures*	GluA2	Activity deprivation (TTX 48 h) Activity elevation (BIC + 4-AP 48 h)	Epilepsy (Kaalund et al., 2014) Stress-related disorders (Torres-Berrío et al., 2017) Rett syndrome (Cheng et al., 2014)
Cytoskeleton dynamics and trafficking	miR-310 (Tsurudome et al., 2010) *Drosophila NMJ*	Khc-73	GluRII mutant	
	miR-132 (Mellios et al., 2011; Tognini et al., 2011) *Mouse visual cortex*	P250GAP	Monocular deprivation	Huntington (Packer et al., 2008) Alzheimer (Lau et al., 2013; Patrick et al., 2017) Autism spectrum disorder (Abu-Elneel et al., 2008; Talebizadeh et al., 2008; Sarachana et al., 2010) Epilepsy (Peng et al., 2013) Schizophrenia (Kim et al., 2010)
RNA-binding proteins	miR-134 (Fiore et al., 2014) *Rat hippocampal cultures*	Pumilio-2	Activity elevation (PTX 48 h)	Epilepsy (Peng et al., 2013) 22q11.2 deletion syndrome (Stark et al., 2008) Rett syndrome (Urdinguio et al., 2010; Cheng et al., 2014)
	miR-129-5p (Rajman et al., 2017) *Rat hippocampal cultures*	Rbfox1 *Atp2b4 Dcx*	Activity elevation (PTX 48 h)	Alzheimer (Lau et al., 2013; Patrick et al., 2017) Autism spectrum disorder (Abu-Elneel et al., 2008)
VGCCs	miR-103 (Favereaux et al., 2011) *Rat spinal chord neurons*	Cav1.2	Neuropathic rats	Alzheimer (Yang et al., 2018) Chronic Pain (Favereaux et al., 2011) Autism spectrum disorder (Sarachana et al., 2010) Traumatic brain injury (Redell et al., 2009) 22q11.2 deletion syndrome (Stark et al., 2008)

## miRNA-Dependent Control of Post-Synaptic Function During Homeostatic Synaptic Plasticity

One parameter that is commonly regulated to maintain synaptic homeostasis is the abundance of post-synaptic receptors. At excitatory synapses, the accumulation or depletion of synaptic AMPA-type glutamate receptors (AMPARs) has been well-characterized, mostly in primary neuronal cultures, following prolonged deprivation or elevation of neuronal activity, respectively. Depending on how neuronal activity is altered, this plasticity can be cell-wide or synapse-specific, and can engage different signaling pathways and combinations of AMPAR subunits (Vitureira and Goda, 2013; Fernandes and Carvalho, 2016). Interestingly, global pharmacological manipulations of neuronal activity known to induce HSP (Turrigiano et al., 1998; Thiagarajan et al., 2005; Sutton et al., 2006) alter the expression of several miRNAs in primary hippocampal cultures, which likely contribute to the proteome remodeling observed upon such conditions (Schanzenbächer et al., 2016, 2018).

### Homeostatic Increase of Post-synaptic Strength in Response to Activity Deprivation

In rat cultured hippocampal neurons, the blockade for > 4 h of action potentials (APs) and NMDA receptors (NMDARs) with tetrodotoxin (TTX) and APV, respectively, leads to the local synthesis and synaptic insertion of AMPARs likely formed of GluA1 homomers (Sutton et al., 2006). This process is mediated by a decrease of miR-92a targeting the AMPAR subunit GluA1 in dendrites (Letellier et al., 2014). As a result, GluA1 translation is de-repressed and new AMPARs are targeted to synapses to support the increase in synaptic strength (Letellier et al., 2014). Importantly, this form of HSP is maintained in dendrites disconnected from the cell body (Sutton et al., 2006; Letellier et al., 2014), suggesting that transcription is not required and that the miR-92a-dependent GluA1 translation occurs locally. Intriguingly, incubating hippocampal neurons with TTX/APV for longer periods (>12 h) increases the expression of another miRNA, miR-124, which targets the GluA2 AMPAR subunit (Ho et al., 2014; Hou et al., 2015). While both miR-92a downregulation and miR-124 upregulation promote the expression of GluA2-lacking, calcium permeable AMPARs, the TTX/APV-induced elevation of miR-124 seems to rely on transcription-dependent mechanisms (Hou et al., 2015) and therefore may affect synaptic strengths more widely and uniformly as compared to miR-92a.

Interestingly, a 24 h activity-deprivation paradigm in cultured hippocampal neurons using non-competitive antagonists of AMPARs and NMDARs (GYKI-52466 and MK-801, respectively) does not affect miR-92a or miR-124 levels but rather downregulates miR-186-5p, a miRNA which also targets GluA2, thereby leading to the synaptic insertion of GluA2-containing AMPARs which are not permeable to calcium (Silva et al., 2019). Finally, a 24–48 h treatment with TTX alone induces the insertion of GluA2-containing AMPARs (Sutton et al., 2006; Gainey et al., 2009), but a specific regulation of this process by miRs has not been reported yet. Together, these studies suggest that neurons engage different miR-dependent pathways depending on the activity-deprivation paradigm, to produce a selective homeostatic compensation with regards to the AMPAR subunit composition that confers specific plastic properties to synapses (Diering and Huganir, 2018). A key point will be to determine the functional significance of these multiple miRNA-dependent regulations, and whether they extend to more physiological –*in vivo*- systems and to other brain regions.

### Homeostatic Decrease of Post-synaptic Strengths in Response to Activity Elevation

The expression of some specific miRNAs is also altered following pharmacological manipulations to elevate network activity (Fiore et al., 2014; Rajman et al., 2017; Rocchi et al., 2019), suggesting that miRNAs bi-directionally adapt synaptic strengths across dendrites depending on network activity. In cultured hippocampal neurons, miR-134 elevation induced by the chronic (>24 h) pharmacological blockade of GABA_A_ receptors (GABA_A_Rs) using picrotoxin (PTX) contributes to homeostatic synaptic downscaling by decreasing GluA2 surface expression and by promoting the elimination of excitatory synapses (Fiore et al., 2014). Specifically, miR-134 downregulates the RNA-binding protein Pumilio 2 which normally inhibits the polo-like kinase 2 (Plk2) pathway that promotes homeostatic downscaling through the degradation of the spine-associated protein RapGAP SPAR (SPAR) and the sequestration of the GluA2-interacting *N*-ethylmaleimide-sensitive fusion (NSF) protein (Seeburg et al., 2008; Evers et al., 2010). Curiously, other known targets of miR-134 including the protein kinase Limk1 which promotes spine development by regulating actin dynamics (Schratt et al., 2006) are not affected by the PTX treatment (Fiore et al., 2014), suggesting a selective effect. Interestingly, miR-134 is also upregulated in the temporal lobe neocortex of patients with epilepsy (Jimenez-Mateos et al., 2012). While it is currently unknown whether Pumilio 2 is downregulated in this condition, Limk1 expression level is decreased, which could result in smaller dendritic spines to dampen hyperactivity and may represent some homeostatic adaptation (Jimenez-Mateos et al., 2012). Surprisingly, however, silencing miR-134 in mice using antagomirs suppresses seizures and has a neuroprotective action (Jimenez-Mateos et al., 2012), suggesting that abnormal increased levels of miR-134 may rather promote epilepsy. Therefore, despite the therapeutical potential of miR-134 antagomirs in the context of epilepsy, more investigations are required to understand the exact mode of action of miR-134 *in vivo*.

In another study, miR-129-5p elevation was also shown to be required for the PTX-induced downscaling of synaptic strength, by promoting the downregulation of the calcium pump Atp2b4 and the microtubule-associated protein doublecortin (Dcx) (Rajman et al., 2017). Furthermore, the authors uncover a functional interaction between miR-129-5p and the RNA binding protein Rbfox1, which normally promotes the expression of both Atp2b4 and Dcx through their 3′ UTR. Upon PTX-induced synaptic scaling, Rbfox1 expression is downregulated in a miR-129-5p manner, thereby allowing the repression of Atp2b4, Dcx and possibly other synaptic genes. However, how miR-134 and miR-129-5p work in conjunction to drive homeostatic downscaling triggered by GABAR blockade and whether other regulated miRNAs including miR-132, miR-495, miR-543-3p, or miR-218 contribute to this process (Rajman et al., 2017; Rocchi et al., 2019) remain to be investigated.

## miRNA-Dependent Control of Presynaptic Function During Homeostatic Synaptic Plasticity

While many studies have uncovered post-synaptic mechanisms for homeostatic synaptic plasticity, there is strong evidence that neurons can also regulate the number and efficacy of presynaptic release sites to compensate for prolonged perturbations in network activity (Thiagarajan et al., 2005; Jakawich et al., 2010; Lindskog et al., 2010; Vitureira et al., 2011; Davis and Müller, 2015). Several miRNAs likely contribute to this process by targeting presynaptic proteins. For instance, in cultured hippocampal neurons, miR-485 is upregulated following the chronic elevation of neuronal activity (>5 days) using bicuculline and 4-aminopyridine (4-AP) to block GABA_A_Rs and potassium channels, respectively (Cohen et al., 2011). miR-485 targets the synaptic vesicle protein 2A (SV2A) which is known to facilitate neurotransmitter release through an interaction with synaptotagmin (Custer, 2006; Yao et al., 2010), and which is downregulated following seizures in the hippocampus, thus possibly representing a homeostatic mechanism (van Vliet et al., 2009). Surprisingly, miR-485 expression does not downregulate presynaptic neurotransmitter release *per se* but rather decreases the number of functional synapses, as evidenced by a decreased density of PSD-95 and AMPAR clusters, suggesting a functional crosstalk between pre and post-synaptic elements. In any case, the mechanism by which miR-485 adapts the number of functional synapses in response to elevated network activity (and possibly during epilepsy) remains to be clarified, as other targets of miR-485 may also contribute to this process (Cohen et al., 2011).

One model system that has been extensively studied in the context of presynaptic homeostatic plasticity is the drosophila neuromuscular junction, where experimentally reducing the sensitivity or the expression of post-synaptic glutamate receptors is precisely balanced by an increase in glutamate release through retrograde signaling (Petersen et al., 1997; Frank et al., 2006; Frank, 2014; Davis and Müller, 2015). The miR-310-313 cluster contributes to this process most likely by targeting the kinesin family member khc-73 in motor neurons (Tsurudome et al., 2010). Specifically, overexpressing miR-310 or knocking-down khc-73 in motor neurons both inhibit the homeostatic increase in quantal content normally observed in *GluRIIA* mutants.

miR-1000 is another drosophila miRNA which modulates glutamate release by down-regulating the expression of the glutamate transporter VGlut (Verma et al., 2015). miR-1000 genetic deletion enhances VGlut expression, resulting in an excess of glutamate release through a higher number of active boutons, which are also bigger in size. Interestingly, miR-1000 expression level in the drosophila visual system is regulated in an homeostatic manner by visual input. Indeed, miR-1000 transcript levels are significantly reduced in dark-reared flies, raising the possibility that glutamate release is enhanced and compensates for reduced sensory input. In contrast, flies reared in constant light show increased miR-1000 expression compared to animals reared under a normal light-dark cycle, suggesting a reduction of glutamate release to compensate for a prolonged elevation of sensory input (Verma et al., 2015). Importantly, the failure of miR-1000-dependent regulation of glutamate release results in excitotoxicity and reduced neuron survival. While miR-1000 is not expressed in mammals, the seed-similar miRNA miR-137 is expressed in mouse hippocampal neurons and may similarly regulate VGluT2 (Verma et al., 2015) in addition to its post-synaptic target GluA1 (Olde Loohuis et al., 2015). Interestingly, miR-137 has been genetically associated with schizophrenia and miR-137 overexpression in the mouse dentate gyrus impairs presynaptic plasticity and hippocampus-dependent learning and memory through the regulation of the presynaptic proteins synaptotagmin-1, complexin-1, and NSF (Siegert et al., 2015).

## miRNA-Dependent Control of Experience-Dependent Homeostatic Synaptic Plasticity *In Vivo*

Besides compensating for global perturbations of network activity, whether induced pharmacologically or genetically (see above), there is evidence that HSP also contributes to experience-dependent plasticity and refinement of developing synaptic circuits. In such situations, the strengthening of active inputs is compensated by the weakening of less active inputs on the target cell, presumably through competition-based mechanisms; this eventually leads to the selective stabilization of specific inputs at the expense of others. This activity-dependent process has been extensively studied in the mammalian visual cortex where a population of neurons respond to the two eyes. Occluding the vision of one eye during a critical developmental period (monocular deprivation paradigm), produces a loss of responsiveness of binocular neurons to the deprived eye which is precisely balanced by a corresponding homeostatic increase in response to the undeprived eye (ocular dominance shift), thus preserving the net visual drive for each neuron (Mrsic-Flogel et al., 2007; Kaneko et al., 2008; Ranson et al., 2012; Kaneko and Stryker, 2017).

Interestingly, the expression of some specific miRNAs is altered following sensory deprivation in the visual cortex, and may contribute to the homeostatic component of the ocular dominance shift. Among them, miR-132, is decreased after monocular deprivation or dark rearing (Mellios et al., 2011; Tognini et al., 2011). Inhibiting miR-132 through the injection of a miRNA-sponge-expressing lentivirus (Mellios et al., 2011) or counteracting miR-132 reduction by infusing a miR-132 mimic (Tognini et al., 2011) both prevent the ocular dominance plasticity shift induced by monocular sensory deprivation, suggesting that miR-132 drop is necessary to both weaken the deprived visual input and strengthen the undeprived input. At the cellular level, such a homeostatic balance between active and inactive inputs may involve the de-repression of the miR-132 target p250GAP, a Rho family GTPase that regulates spine morphology and remodeling through Rac1 inhibition (Wayman et al., 2008; Edbauer et al., 2010; Impey et al., 2010; Remenyi et al., 2013) and which has been implicated in epileptogenesis process (Yuan et al., 2016). In one possible mechanism, sensory-deprived synaptic inputs could depress and shrink through the GTPase p250GAP/Rac1 pathway while more active synapses would get strengthened and grow in size. Importantly, the differential regulation of active versus inactive synapses in the same post-synaptic cell suggests the existence of local signaling within dendrites (Oh et al., 2015; El-Boustani et al., 2018; Letellier et al., 2019) to which miRNAs might contribute.

## miRNA-Dependent Control of Intrinsic Excitability

In addition to controlling synapse number and efficacy to compensate for local or global activity perturbations, miRNAs can directly regulate membrane excitability, thereby controlling the probability that synaptic inputs trigger action potentials in dendrites and/or axon. For instance, miR-128, which is highly abundant in the mammalian brain, regulates neuronal excitability and motor behavior in the mouse by downregulating the expression of various ion channels and signaling components of the extracellular signal-regulated kinase ERK2 network (Tan et al., 2013). Interestingly, a reduction in miR-128 expression causes increased motor activity and fatal epilepsy in mice. While it would be interesting to see to what extent variations in network activity affect miR-128 expression, these finding suggests that the level of miR-128 is tightly regulated to maintain the neuronal firing rate (Tan et al., 2013).

miRNAs have been involved in the control of intrinsic excitability through the regulation of voltage-gated calcium channel. In the context of chronic pain, miR-103 regulates the expression of the three subunits of the Cav1.2-comprising L-type calcium channel in rat spinal cord neurons, thereby modulating sensitization to pain. Moreover, miR-103 was downregulated in neuropathic rats and miR-103 intrathecal applications successfully relieved pain, thus identifying miR-103 as a possible therapeutic target in neuropathic chronic pain (Favereaux et al., 2011).

Another example is miR-129 which not only controls homeostatic downscaling by targeting Atp2b4 and Dcx (see above; Rajman et al., 2017) but also regulates the dendritic expression of the *Shaker*-like potassium channel Kv1.1 (Sosanya et al., 2013). Kv1.1 is a dendrotoxin-sensitive voltage gated potassium channel that is expressed in the axon but also in dendrites (Raab-Graham et al., 2006; Sosanya et al., 2013). A proposed mechanism involves miR-129 and the mRNA binding protein HuD which binds to Kv1.1 mRNA, depending on mTORC1 kinase activity to repress or enhance Kv1.1 expression, respectively (Sosanya et al., 2013). Interestingly, miR-129-mediated translational repression of Kv1.1 is enhanced 3 weeks after *status epilepticus* in rats, suggesting that miR-129 promotes excitability by targeting Kv1.1 and that this mechanism is tightly regulated to maintain neuronal homeostasis (Sosanya et al., 2015). However, that the same miRNA can promote both synaptic downscaling and dendritic excitability suggests the involvement of complex regulations to orchestrate the homeostatic response in time and space.

There is also evidence that the RNA-binding protein Pumilio 2, a key miR-134 target involved in PTX-induced downscaling (see above), controls the homeostasis of membrane excitability in cultured cortical neurons. Indeed, Pumilio 2 expression at the cellular level is increased upon elevating neuronal activity and thereby suppresses translation of the voltage-gated sodium channel transcript Nav1.6 to decrease intrinsic excitability (Driscoll et al., 2013). However, this is at odds with the fact that the prolonged elevation of neuronal activity reduces Pumilio 2 expression locally in the dendritic compartment in a miR-134-dependent manner to promote downscaling (Fiore et al., 2014). Therefore, it is unclear how elevating activity can simultaneously promote the up- and down-regulation of Pumilio 2 to cause decrease in membrane excitability and miR-134-dependent synaptic downscaling, respectively. This suggests the existence of compartmentalized mechanisms, where Pumilio 2 expression might be differently regulated in the cell body versus dendrites, but this remains to be investigated experimentally.

## Do miRNAs Regulate Homeostatic Plasticity Locally?

What makes miRNAs interesting candidates in the regulation of synaptic plasticity is that they potentially control protein synthesis in remote subcellular compartments such as dendrites and synapses to provide an appropriate and targeted physiological response. While this idea has not been directly tested in the context of HSP, some of the studies discussed above provide indirect evidence that miRNA-dependent homeostatic plasticity requires local regulations, supporting the concept that synapses do not always adapt uniformly and that homeostatic plasticity can operate within autonomous subcellular compartments, and down to single synapses (Thiagarajan et al., 2005; Sutton et al., 2006; Echegoyen et al., 2007; Aoto et al., 2008; Branco et al., 2008; Kim and Tsien, 2008; Maghsoodi et al., 2008; Beique et al., 2011; Wang et al., 2019).

In support of a role for miRNAs in regulating the function of local compartments like synapses, subcellular fractionation and *in situ* hybridization experiments revealed that several miRNAs are present in dendrites, axons or even synapses and that neuronal activity regulates both their abundance and function (Kye et al., 2007; Lugli et al., 2008; Schratt, 2009; Siegel et al., 2009; Natera-Naranjo et al., 2010). Interestingly, the distribution of miRNAs seems to parallel the distribution of their cognate target mRNAs (Kye et al., 2007); such a spatial proximity may enable the efficient regulation of local protein translation to serve a specific function at the right time and place (Kosik, 2016; Park et al., 2019).

What are the mechanisms by which neuronal activity regulates the local amount of miRNAs? While the activity-dependent expression of several miRNAs including miR-132, miR-134 and miR-124 may be regulated at the transcriptional level by transcription factors such like CREB, Mef2 or EVI1 (Fiore et al., 2009; Nudelman et al., 2010; Remenyi et al., 2010; Hou et al., 2015), there is evidence that neuronal activity directly controls the local processing of pre-miRNAs into mature miRNAs at the level of single dendritic spines. Using a fluorescent pre-miRNA sensor to probe Dicer activity, it was recently shown that the local stimulation of single spines through glutamate uncaging promotes the maturation of miR-181a in a NMDAR-dependent manner, leading to the local repression of CamKIIα synthesis (Sambandan et al., 2017). Furthermore, the local abundance of miR-134, previously implicated in PTX-induced downscaling (Fiore et al., 2014), varies depending on spine maturation and activity, while BDNF local stimulation leads to a decrease in the number of miR-134 copies present at the neck of spines (Park et al., 2019). In addition to the local control of miRNA maturation through Dicer, neuronal activity regulate the turnover of the RISC complex itself, which could possibly impact miR-dependent local protein translation in a non-specific way. In particular, the RISC component MOV10 is degraded upon NMDAR activation, which may result in the release of miRNAs from their mRNA targets and de-repress local protein translation (Chendrimada et al., 2007; Banerjee et al., 2009).

However, one important question remains: how specific activity variations can regulate the local expression and/or function of some specific miRNAs and not others in order to achieve the appropriate physiological response? Potential mechanisms involve specific interactions with cognate mRNA targets which could protect miRNAs from degradation (Pitchiaya et al., 2017), storage in P-bodies whose dendritic location is regulated by neuronal activity (Cougot et al., 2008), or interaction with circular RNAs serving as natural miRNA-sponges (Hansen et al., 2013).

## Conclusion

There is strong evidence that miRNAs contribute to homeostatic plasticity and associated neurological disorders including epilepsy, neuropsychiatric, and neurodegenerative diseases (Mellios and Sur, 2012; Henshall et al., 2016; Quinlan et al., 2017; Rajman et al., 2017) (Table 1). However, despite some recent progress, important questions remain. In particular, the signaling pathways linking physiological synaptic activity variations to miRNA function, trafficking, and turnover remain largely unknown, as most of the current knowledge relies on pharmacological manipulations in culture systems. Some effort will thus be required to investigate the role of identified miRNAs in more physiologically relevant systems; the development of new probes and live-imaging tools to track individual RNAs and investigate translation dynamics (Park et al., 2014; Wang et al., 2016; Wu et al., 2016; Yan et al., 2016) should provide new insights into these mechanisms. Equally important will be to investigate whether they regulate inhibitory synapses which also undergo homeostatic plasticity (Kilman et al., 2002; Peng et al., 2010; Rannals and Kapur, 2011), and whether they contribute to the neuron-glia interactions involved in homeostatic plasticity (Stellwagen and Malenka, 2006; Letellier et al., 2016). Finally, considering that miRNAs also control LTP and depression (Hu and Li, 2017), it will be interesting to investigate whether and how miRNAs enable the integration in time and space of both Hebbian and homeostatic plasticity. A better understanding of the miRNA function in synaptic plasticity and the possible links with pathologies will be very helpful in refining promising therapeutic strategies (Wen, 2016; Angelucci et al., 2019).

## Author Contributions

ML, OT, AF, and SD wrote the manuscript. SD built the figure.

## Conflict of Interest

The authors declare that the research was conducted in the absence of any commercial or financial relationships that could be construed as a potential conflict of interest.
